# A model based on adipose and muscle-related indicators evaluated by CT images for predicting microvascular invasion in HCC patients

**DOI:** 10.1186/s40364-023-00527-z

**Published:** 2023-10-04

**Authors:** Xin-Cheng Mao, Shuo Shi, Lun-Jie Yan, Han-Chao Wang, Zi-Niu Ding, Hui Liu, Guo-Qiang Pan, Xiao Zhang, Cheng-Long Han, Bao-Wen Tian, Dong-Xu Wang, Si-Yu Tan, Zhao-Ru Dong, Yu-Chuan Yan, Tao Li

**Affiliations:** 1 Department of General Surgery, Qilu Hospital, Shandong University, 107 West Wen Hua Road, Jinan, 250012 China; 2 Department of Radiology, Qilu Hospital, Shandong University, Jinan, China; 3 Institute for Financial Studies, Shandong University, Jinan, 250100 China

**Keywords:** Hepatocellular carcinoma, Microvascular invasion, Adipose and muscle tissues, Computed tomography, Nomogram, Survival analysis

## Abstract

**Background and aim:**

The presence of microvascular invasion (MVI) will impair the surgical outcome of hepatocellular carcinoma (HCC). Adipose and muscle tissues have been confirmed to be associated with the prognosis of HCC. We aimed to develop and validate a nomogram based on adipose and muscle related-variables for preoperative prediction of MVI in HCC.

**Methods:**

One hundred fifty-eight HCC patients from institution A (training cohort) and 53 HCC patients from institution B (validation cohort) were included, all of whom underwent preoperative CT scan and curative resection with confirmed pathological diagnoses. Least absolute shrinkage and selection operator (LASSO) logistic regression was applied to data dimensionality reduction and screening. Nomogram was constructed based on the independent variables, and evaluated by external validation, calibration curve, receiver operating characteristic (ROC) curve and decision curve analysis (DCA).

**Results:**

Histopathologically identified MVI was found in 101 of 211 patients (47.9%). The preoperative imaging and clinical variables associated with MVI were visceral adipose tissue (VAT) density, intramuscular adipose tissue index (IMATI), skeletal muscle (SM) area, age, tumor size and cirrhosis. Incorporating these 6 factors, the nomogram achieved good concordance index of 0.79 (95%CI: 0.72–0.86) and 0.75 (95%CI: 0.62–0.89) in training and validation cohorts, respectively. In addition, calibration curve exhibited good consistency between predicted and actual MVI probabilities. ROC curve and DCA of the nomogram showed superior performance than that of models only depended on clinical or imaging variables. Based on the nomogram score, patients were divided into high (> 273.8) and low (< = 273.8) risk of MVI presence groups. For patients with high MVI risk, wide-margin resection or anatomical resection could significantly improve the 2-year recurrence free survival.

**Conclusion:**

By combining 6 preoperative independently predictive factors of MVI, a nomogram was constructed. This model provides an optimal preoperative estimation of MVI risk in HCC patients, and may help to stratify high-risk individuals and optimize clinical decision making.

**Supplementary Information:**

The online version contains supplementary material available at 10.1186/s40364-023-00527-z.

## Introduction

Hepatocellular carcinoma (HCC) represents the most frequent type of primary liver cancer and ranks as the third leading cause of cancer-related death worldwide [1]. Surgical resection is the backbone of curative treatment for HCC. Although the surgical operation of HCC has been extremely developed, the prognosis of HCC is still very poor [2]. The recurrence after operation remains a major obstacle to improve survival and the 5-year recurrence rates are up to 70%, even in patients with single small HCC (≤ 2 cm) [3]. One of the main reasons is microvascular invasion (MVI). MVI, a histopathological feature of micro-metastases, can be observed in 15.0%-57.1% of surgical specimens and stands as a key predictor for early recurrence and poor prognosis [4, 5]. In addition, the presence of MVI has been an essential factor influencing the long-term prognosis of HCC patients underwent liver transplantation [6]. However, the impact of MVI has largely been disregarded by clinicians as it can only be found by histologic examination of postoperative specimens. As MVI represents an aggressive behavior of HCC [5], individualized surgical management for patients should be formulated according to the risk–benefit assessment of MVI before surgery. For patients with high risk of MVI, anatomical subsegmentectomy or partial hepatectomy with wide margin is recommended under better liver function, which could decrease the risk of recurrence [7]. Therefore, establishing a highly accurate risk model for predicting MVI can help to stratify high-risk individuals, optimize clinical decision making and improve prognosis.

Over the last decades, numerous efforts have been taken to estimate MVI prior to surgery. Cucchetti et al. utilized four clinical common variables, including alpha-fetoprotein (AFP), tumor number, size and volume, to develop an artificial neural network (ANN) model that accurately identified 91% of MVI cases in testing group [8]. However, this ANN model requires specialized computer software, which potentially limits its utility in routine clinical application. In contrast, the nomogram might be a convenient option and provide highly accurate and individualized risk estimate [9]. For hepatitis B virus–related HCC patients, Li et al. constructed a nomogram by incorporating contrast-enhanced magnetic resonance imaging (MRI) data with hematology data, which achieved relatively good predictive accuracy for MVI [10]. Unfortunately, serum biomarkers might also have abnormally change in non-cancerous liver lesions, so specificity is not optimal. As technologies advance, radiomics nomograms based on contrast-enhanced computed tomography (CT) or gadoxetic acid-enhanced MRI have achieved excellent concordance indexes (C indexes) in predicting MVI [11, 12]. However, random split sample approach was used in these studies to generate training and validation sets. Although this method is commonly used, it represents an inefficient use of the data and has large variability, especially in condition of small population [13]. In addition, the number of candidates radiomic features is orders of magnitude higher than the number of cases, which could lead to overfitting errors [14]. Taken together, it is necessary to identify novel predictors and employ appropriate statistical method to construct a nomogram for predicting MVI before surgery.

Recently, the impact of body composition changes, which mainly encompasses adipose and muscle tissues, on the prognosis of HCC has received wide attention [15–20]. Skeletal muscle index (SMI) [15, 20], subcutaneous adipose tissue index (SATI) [16, 18] and visceral adipose tissue index (VATI) [19, 20] have been recognized as strongly correlated variables with the occurrence and development of HCC. Some of them act as negative factors by increasing inflammatory response, altering the hormonal level or disrupting the balance between anabolic and catabolic metabolisms [21, 22], while others act as protective factors by stimulating the insulin response, boosting lipid metabolism or enhancing immune function [16]. Therefore, it’s speculated that the changes of adipose and muscle tissues may have some correlation with the presence of MVI in HCC patients. In fact, Arsenii et al. [23] and Wu et al. [24] have performed relevant studies. Despite their efforts, these studies lacked sufficient evidence, as the former did not yield any positive results, while the latter only proved the correlation of SAT and VAT with the presence of MVI in univariate logistic analysis. In this study, we aim to collect adipose and muscle related clinical variables of HCC patients to determine the variables that are most strongly associated with MVI, and construct a nomogram model to assist surgeons in risk stratification and personalized treatment.

## Methods

### Study population

Between January 01, 2018, and December 31, 2021, retrospective data on patients diagnosed with HCC through postoperative pathology were collected at Qilu hospital of Shandong university (institution A) and the Second Hospital of Shandong University (institution B). This retrospective study was reviewed and approved by Institutional Review Board of Qilu hospital of Shandong university [approval number: 2021(222)].

**Fig. 1:**
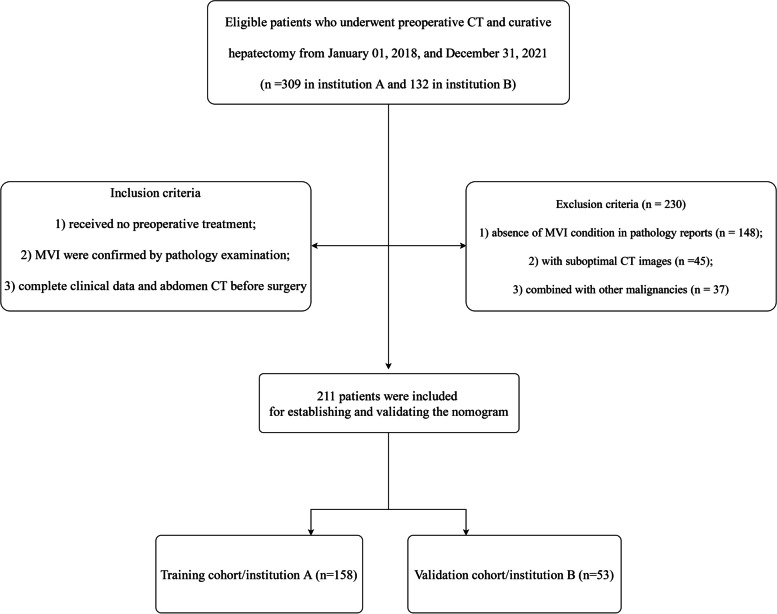
Flowchart of the included HCC patients

Inclusion criteria were as follows: 1) liver resection was the initial treatment, and patients received no preoperative treatment, such as transcatheter arterial chemoembolization (TACE), targeted immunotherapy, or radiotherapy; 2) the diagnosis of MVI was confirmed by pathological examination; 3) complete clinical data can be acquired from electronic medical record, and CT scan of the abdomen was performed within two weeks before surgery in these two hospitals. Patients with a history of other malignancies, suboptimal CT images, absence of MVI condition in pathology reports were excluded. The detailed screening procedure was shown in Fig. 1.

### Clinical data and follow-up

Clinical data included the MVI status (negative vs. positive), age (< 60 vs. ≥ 60 years), sex (female vs. male), tumor size (< 5 vs. ≥ 5 cm), Child–Pugh class (A vs. B), cirrhosis (no vs. yes), hepatitis B surface antigen (HBsAg) status (negative vs. positive), albumin (ALB, < 3.5 vs. ≥ 3.5 g/dL), alpha fetoprotein (AFP, < 400 vs. ≥ 400 ng/mL), aspartate transaminase (AST, < 40 vs. ≥ 40 U/L) level, alanine aminotransferase (ALT, < 44 vs. ≥ 44 U/L) level, total bilirubin (TB, < 17.1 vs. ≥ 17.1 µmol/L) level, body mass index (BMI, < 24.9 vs. ≥ 24.9 kg/m^2^), surgical approach (open laparotomy vs. laparoscopic), resection method (non-anatomical vs. anatomical), and surgical margin (< 1 vs. ≥ 1 cm). The definition of surgical margin was the shortest distance from the edge of the tumor to the resection line. MVI is defined as the presence of cancer cell clusters within the lumen of blood vessels lined by endothelial cells under a microscope [4]. As macrovascular wall contains layers of smooth muscle or elastic fibers, while microvascular wall typically consists only of endothelial cells. In order to help distinguish MVI from macrovascular invasion, immunohistochemistry was performed to examine the expression of CD34 (vascular endothelium), smooth muscle α-actin (vascular smooth muscle), and elastic fibers (elastic fiber layer of tiny blood vessel wall) [25].

All patients were recommended to surveillance every 3 months for the first 2 years and then every 6 months thereafter. Postoperative follow-up included abdominal ultrasound, CT or MRI, routine blood, serum biochemistry, and tumor-specific biomarkers. Early recurrence was defined as intrahepatic recurrence within two years after curative resection. Recurrence free survival (RFS) was defined as the duration from the data of surgery to either the date of tumor recurrence or the last follow-up. Overall survival (OS) was defined as the interval from the date of surgery to the date of death or the last follow-up.

### CT-related body composition variables

Abdominal CT scan for included patients from these two hospitals were performed within two weeks before surgery using the equipment of Siemens 64-slice spiral CT scanner from the same manufacturer [26, 27] to avoid data heterogeneity resulting from CT equipment. Semi‐automatic software (SliceOmatic™ version 5.0, Tomovision, Montreal, Quebec, Canada) was utilized to quantify the areas and density of skeletal muscle (SM) tissue and different adipose tissues [(i.e., subcutaneous adipose tissue (SAT), visceral adipose tissue (VAT) and intramuscular adipose tissue (IMAT)] in the single cross-sectional CT images at the central level of the third lumbar vertebra (L3). The areas quantified from the single cross-sectional CT image at the L3 level exhibited the highest correlation with the overall body tissue mass, demonstrating correlation coefficients (r^2^) of 0.855 and 0.927 for muscle and adipose tissues, respectively [28]. The SM tissue at L3 level consisted of the psoas, quadratus lumborum, erector spinae, transversus abdominis, external and internal obliques, and rectus abdominis muscles. SM area referred the sum of cross‐sectional areas of the aforementioned muscles, measured in centimeters squared (cm^2^). Area of each adipose tissues also referred the cross-sectional area on the L3 level. The above each tissue areas were normalized by the heights squared (m^2^) to obtain their corresponding indexes (cm^2^/m^2^), including SMI, SATI, VATI and IMAT index (IMATI). Density of muscle and adipose tissues referred the mean radiologic tissue attenuation for each type of tissue, measured in Hounsfield Unit (HU). The formula for calculating visceral to subcutaneous adipose tissue area ratio (VSR) was VAT area divided by SAT area. The specific steps to obtain body composition-related parameters were as follows: Firstly, the radiodensity threshold-based technique of SliceOmatic™ was utilized to semi-automatically outline the initial delineations of the region of interest. The HU thresholds were applied as follows: -30 to 150 HU for SM, -190 to -30 HU for SAT and IMAT, and -150 to -30 HU for VAT [29, 30]. Then a clinician under the guidance of an experienced radiologist performed a manual inspection and adjustment of the region of interest without knowing the patients’ characteristics and outcomes. Finally, the areas (cm^2^) and density values (HU) were acquired to provide characterization for each body composition. Examples of delineation were shown in the Supplementary Fig. 1. The "SurvivalROC" package of R software was used to calculate the cut-off values for each variable. This package could repeatedly evaluate cut-off values obtained from receiver operating characteristic (ROC) curves and identified the optimal cut-off values based on the maximum log-rank statistic. Notably, cut-off values were also determined according to gender, as body compositions varied between genders. The detailed cut-off values were shown in Supplementary Table 1. To ensure the inter-reader consistency, two clinicians with more than 5 years’ experience in hepatobiliary surgery independently completed the delineations of all variables. Then 40 pairs of samples were randomly selected for comparison and the inter-reader fluctuated between 0.87 and 0.91.

### Statistical analysis

Chi-square test was utilized to compared the categorical variables, and results were presented as absolute counts and percentages format [No. (%)]. The process of data dimensionality reduction and screening was implemented by the least absolute shrinkage and selection operator (LASSO) regression analysis. Variables with non-zero coefficients were first included in univariate logistic regression analysis, and then all variables with *P*-values less than 0.1 were entered into multivariate stepwise forward regression analysis to identify independent variables with *P*-values less than 0.05. A nomogram was constructed using the independent predictors. To assess the predictive performance of the nomogram, we performed calibration curve and C index yielded by 1000 bootstrapping techniques. The clinical utility of nomogram was evaluated through comparing its ROC curve and decision curve analysis (DCA) with those of imaging and clinical model. In addition, we used 200 rounds of tenfold internal cross-validation and external validation methods to evaluate our nomogram. The MVI risk score for all patients was computed using the nomogram, followed by allocation of all patients into low and high-risk groups using the cut-off value with the highest Youden index (the sum of sensitivity and specificity). Finally, the Kaplan–Meier (KM) method and the log-rank test were employed to generate survival curves for the purpose of stratifying risk factors and helping surgeons to make more personalized treatment plans. The above all statistical analyses were performed using and R software (version 4.0.2), with packages: “rms”, “survival”, “pROC”, “caret”, “rmda”.

## Results

### Baseline characteristics of patients

During the study period, 158 HCC patients form institution A and 53 HCC patients form institution B met the inclusion criteria were enrolled. The former was the training cohort with the median follow-up time of 23.9 months, and the latter was the validation cohort with the median follow-up time of 27.1 months. Pathological confirmed MVI was found in 101 (47.9%) individuals of all patients. The detailed clinical, adipose as well as muscle related variables were shown in Table 1, respectively.

**Table 1:** Baseline characteristics of HCC patients

Variable	Cohort, No. (%)			*P* value
	Overall (*n* = 211)	Training cohort	Validation cohort	
		(*n* = 158)	(*n* = 53)	
MVI				0.36
Negative	110 (52.1)	79 (50.0)	31 (58.5)	
Positive	101 (47.9)	79 (50.0)	22 (41.5)	
Sex				0.75
Female	41 (19.4)	32 (20.3)	9 (17.0)	
Male	170 (80.6)	126 (79.7)	44 (83.0)	
Age (years)				0.02
< 60	118 (55.9)	96 (60.8)	22 (41.5)	
> = 60	93 (44.1)	62 (39.2)	31 (58.5)	
Tumor size (cm)				0.05
< 5	98 (46.4)	80 (50.6)	18 (34.0)	
> = 5	113 (53.6)	78 (49.4)	35 (66.0)	
Child–Pugh class				0.99
A	201 (95.3)	150 (94.9)	51 (96.2)	
B	10 (4.7)	8 (5.1)	2 (3.8)	
Cirrhosis				0.96
No	69 (32.7)	51 (32.3)	18 (34.0)	
Yes	142 (67.3)	107 (67.7)	35 (66.0)	
HBsAg				0.34
Negative	44 (20.9)	30 (19.0)	14 (26.4)	
Positive	167 (79.1)	128 (81.0)	39 (73.6)	
Albumin (g/dL)				0.14
< 3.5	13 (6.2)	7 (4.4)	6 (11.3)	
> = 3.5	198 (93.8)	151 (95.6)	47 (88.7)	
AFP (ng/mL)				0.04
< 400	150 (71.1)	106 (67.1)	44 (83.0)	
> = 400	61 (28.9)	52 (32.9)	9 (17.0)	
AST (U/L)				0.75
< 40	145 (68.7)	110 (69.6)	35 (66.0)	
> = 40	66 (31.3)	48 (30.4)	18 (34.0)	
ALT (U/L)				1.00
< 44	146 (69.2)	109 (69.0)	37 (69.8)	
> = 44	65 (30.8)	49 (31.0)	16 (30.2)	
TB (umol/L)				1.00
< 17.1	132 (62.6)	99 (62.7)	33 (62.3)	
> = 17.1	79 (37.4)	59 (37.3)	20 (37.7)	
BMI (kg/m^2^)				0.01
< 24.9	162 (76.8)	135 (85.4)	27 (50.9)	
> = 24.9	49 (23.2)	23 (14.6)	26 (49.1)	
Surgical approach				0.09
Open laparotomy	99 (46.9)	80 (50.6)	19 (35.8)	
Laparoscopic	112 (53.1)	78 (49.4)	34 (64.2)	
Resection method				1.00
Non-anatomical	97 (46.0)	73 (46.2)	24 (45.3)	
Anatomical	114 (54.0)	85 (53.8)	29 (54.7)	
Surgical margin (cm)				0.03
< 1	150 (71.1)	119 (75.3)	31 (58.5)	
> = 1	61 (28.9)	39 (24.7)	22 (41.5)	
SAT area (cm^2^)				1.00
Small	80 (37.9)	60 (38.0)	20 (37.7)	
Large	131 (62.1)	98 (62.0)	33 (62.3)	
SAT density (HU)				0.08
Low	119 (56.4)	95 (60.1)	24 (45.3)	
High	92 (43.6)	63 (39.9)	29 (54.7)	
SATI (cm^2^/m^2^)				0.27
Low	157 (74.4)	114 (72.2)	43 (81.1)	
High	54 (25.6)	44 (27.8)	10 (18.9)	
VAT area (cm^2^)				0.14
Small	88 (41.7)	71 (44.9)	17 (32.1)	
Large	123 (58.3)	87 (55.1)	36 (67.9)	
VAT density (HU)				0.77
Low	90 (55.0)	66 (41.8)	24 (45.3)	
High	121 (45.0)	92 (58.2)	29 (54.7)	
VATI (cm^2^/m^2^)				0.10
Low	90 (42.7)	73 (46.2)	17 (32.1)	
High	121 (57.3)	85 (53.8)	36 (67.9)	
VSR				0.19
Low	90 (42.7)	72 (45.6)	18 (34.0)	
High	121 (57.3)	86 (54.4)	35 (66.0)	
IMAT area (cm^2^)				1.00
Small	107 (50.7)	80 (50.6)	27 (50.9)	
Large	104 (49.3)	78 (49.4)	26 (49.1)	
IMAT density (HU)				0.19
Low	102 (48.3)	81 (51.3)	21 (39.6)	
High	109 (51.7)	77 (48.7)	32 (60.4)	
IMATI (cm^2^/m^2^)				1.00
Low	103 (48.8)	77 (48.7)	26 (49.1)	
High	108 (51.2)	81 (51.3)	27 (50.9)	
SM area (cm^2^)				0.65
Small	63 (29.9)	49 (31.0)	14 (26.4)	
Large	148 (70.1)	109 (69.0)	39 (73.6)	
SM density (HU)				0.55
Low	118 (55.9)	86 (54.4)	32 (60.4)	
High	93 (44.1)	72 (45.6)	21 (39.6)	
SMI (cm^2^/m^2^)				0.40
Low	99 (46.9)	71 (44.9)	28 (52.8)	
High	112 (53.1)	87 (55.1)	25 (47.2)	

*Abbreviations*: *HCC* Hepatocellular carcinoma, *MVI* Microvascular invasion, *HBsAg* Hepatitis B surface Antigen, *AFP* Alpha fetoprotein, *AST* Aspartate aminotransferase, *ALT* Alanine aminotransferase, *TB* Total bilirubin, *BMI* Body mass index, *HU* Hounsfield Unit, *SAT* Subcutaneous adipose tissue, *SATI* Subcutaneous adipose tissue index, *VAT* Visceral adipose tissue, *VATI* Visceral adipose tissue index, *VSR* Visceral to subcutaneous adipose tissue area ratio, *IMAT* Intramuscular adipose tissue, *IMATI* Intramuscular adipose tissue index, *SM* Skeletal muscle, *SMI* Skeletal muscle index

### Data dimensionality reduction and screening

**Fig. 2:**
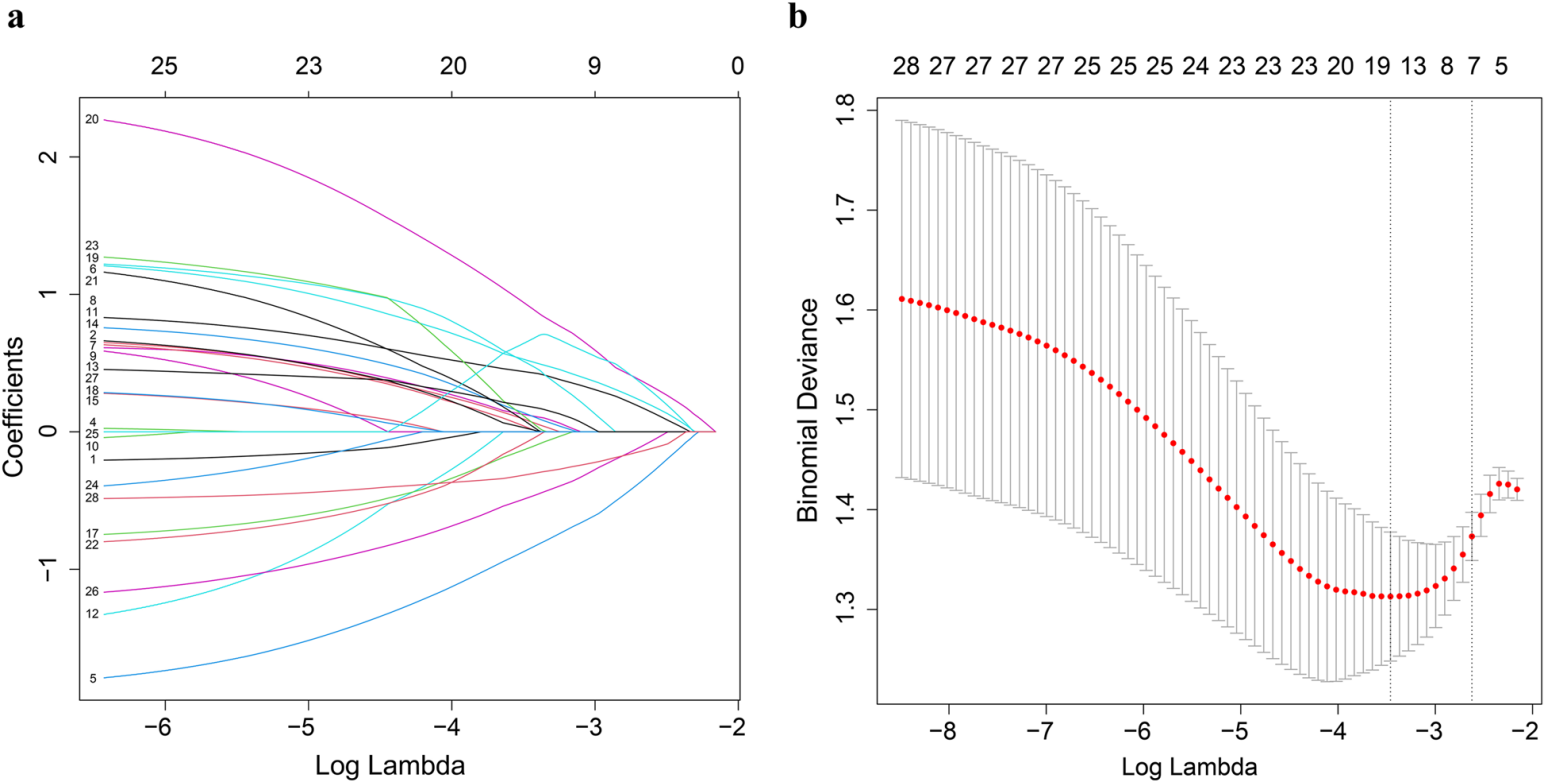
Selecting influence factors by using LASSO regression model. **a** LASSO coefficient curves for 28 predictors. **b** Identification of the best punishment coefficient lambda in LASSO regression model

LASSO regression analysis was performed to identify the most crucial variables linked with the risk of MVI. To guarantee precision, the algorithm's iterations were set to 1000. In addition, cv.glmnet function was used to perform tenfold cross validation to diminish the possibility of overfitting. The distribution of LASSO regression coefficients and cross-validation plots were shown in Fig. 2. Dotted vertical lines indicated the value of lambda.min and lambda.1se. Finally, 18 variables (age, tumor size, CTP, cirrhosis, HBsAg, AFP, TB, resection method, SAT density, VAT area, VAT density, VATI, VSR, IMAT area, IMATI, SM area, SM density, SMI) with nonzero coefficients were selected according to the value of lambda.min (λ = 0.03139931).

### Logistic regression analysis, construction, and validation of nomogram

The above 18 variables were subjected to univariate logistic analysis, and then 8 variables (age, tumor size, cirrhosis, VAT density, IMAT area, IMATI, SM area, SMI) with *P*-values of less than 0.1 were incorporated into multivariate analysis. Except for IMAT area and SMI, the remaining 6 variables [age (OR = 0.214; 95%CI = 0.088–0.522; *P* = 0.001), tumor size (OR = 2.359; 95%CI = 1.115–4.989; *P* = 0.025), cirrhosis (OR = 2.518; 95%CI = 1.077–5.887; *P* = 0.033), VAT density (OR = 3.468; 95%CI = 1.533–7.847; *P* = 0.003), IMATI (OR = 4.783; 95%CI = 2.058–11.112; *P* < 0.001), SM area (OR = 0.295; 95%CI = 0.120–0.725; *P* = 0.008)] were all significant independent predictors for MVI. The detailed data was shown in Table 2.

**Table 2:** Univariate and multivariate logistic analysis of MVI presence for HCC patients

Variable	Univariate Analysis		Multivariable Analysis	
	OR (95% CI)	*P* value	OR (95% CI)	*P* value
Age, > = 60 vs < 60	0.421 (0.216–0.806)	0.010	0.214 (0.088–0.522)	0.001
Tumor size, > = 5 vs < 5, cm	2.274 (1.209–4.336)	0.011	2.359 (1.115–4.989)	0.025
Child–Pugh class, B vs A	3.164 (0.703–22.073)	0.167		
Cirrhosis, yes vs no	2.431 (1.231–4.921)	0.012	2.518 (1.077–5.887)	0.033
HBsAg, positive vs negative	1.958 (0.875–4.568)	0.108		
AFP, > = 400 vs < 400, ng/mL	1.586 (0.815–3.124)	0.177		
TB, > = 17.1 vs < 17.1, umol/L	1.311 (0.688–2.514)	0.411		
Resection method	1.430 (0.764–2.691)	0.265		
SAT density, high vs low	0.620 (0.324–1.175)	0.145		
VAT area, large vs small	1.432 (0.764–2.699)	0.264		
VAT density, high vs low	2.601 (1.365–5.047)	0.004	3.468 (1.533–7.847)	0.003
VATI, high vs low	1.290 (0.690–2.424)	0.425		
VSR, high vs low	0.736 (0.391–1.377)	0.338		
IMAT area, large vs small	2.047 (1.091–3.887)	0.027	-	0.836
IMATI, high vs low	2.158 (1.149–4.107)	0.018	4.783 (2.058–11.112)	< 0.001
SM area, large vs small	0.457 (0.225–0.906)	0.027	0.295 (0.120–0.725)	0.008
SM density, high vs low	1.671 (0.891–3.159)	0.111		
SMI, high vs low	0.460 (0.241–0.866)	0.017	-	0.335

*Abbreviations*: *HCC* Hepatocellular carcinoma, *HBsAg* Hepatitis B surface Antigen, *AFP* Alpha fetoprotein, *TB* Total bilirubin, *SAT* Subcutaneous adipose tissue, *VAT* Visceral adipose tissue, *VATI* Visceral adipose tissue index, *VSR* Visceral to subcutaneous adipose tissue area ratio, *IMAT* Intramuscular adipose tissue, *IMATI* Intramuscular adipose tissue index, *SM* Skeletal muscle, *SMI* Skeletal muscle index

**Fig. 3:**
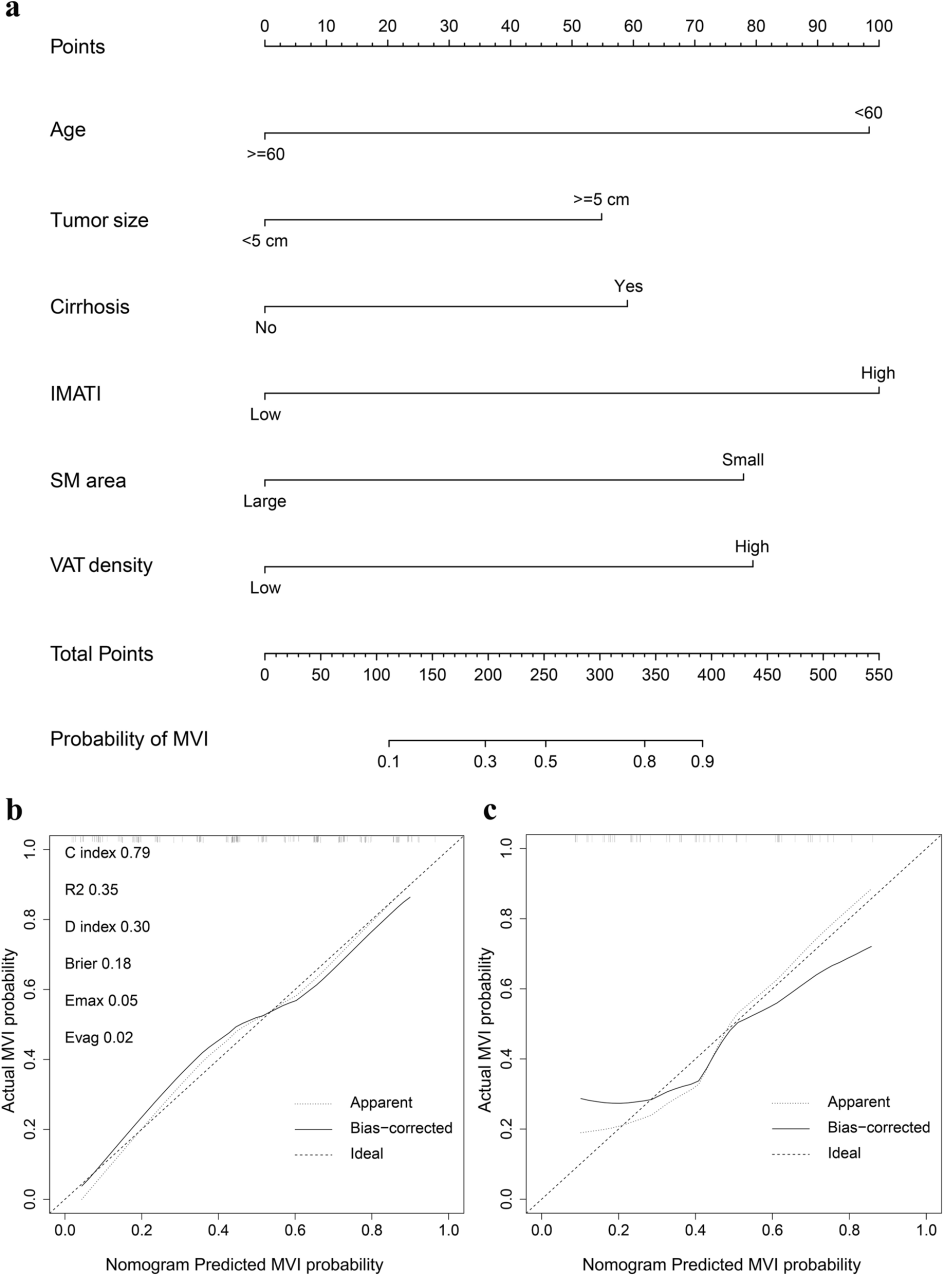
Nomogram model for predicting MVI of HCC patients (**a**) and the calibration curve of the nomogram in training (**b**) and validation cohorts (**c**)

These Six independent predictors were utilized to form an MVI-predicting nomogram (Fig. 3a). The nomogram provided good accuracy in estimating the risk of MVI, with C index of 0.79 (95%CI: 0.72–0.86), R^2^ of 0.35, discrimination index (D index) of 0.30, Brier score of 0.18, Emax of 0.05, Evag of 0.02 (Fig. 3b). Following 200 rounds of tenfold internal cross-validation, there was little variation in the values of the above variables (Table 3), with the correct C index of 0.79, R^2^ of 0.32, D index of 0.24, Brier score of 0.18, Emax of 0.30, Evag of 0.13. In addition, calibration curve exhibited good consistency between predicted MVI probability of nomogram and the actual probability.

**Table 3:** The changes variables after 200 rounds of tenfold internal validation

Variable	Primary value	Calibration value	Change
C index	0.79	0.79	0
R^2^	0.35	0.32	-0.03
D index	0.30	0.24	-0.06
Brier score	0.18	0.18	0
Emax	0.05	0.30	+ 0.25
Eavg	0.02	0.13	+ 0.11

*Abbreviations*: *C index* Concordance index, *D index* Discrimination index

In validation cohort, the nomogram presented a C index of 0.75 (95%CI: 0.62–0.89) for the evaluation of risk associated with MVI. Moreover, the fitting calibration curve was acceptable (Fig. 3c).

### Clinical utility of the nomogram

**Fig. 4:**
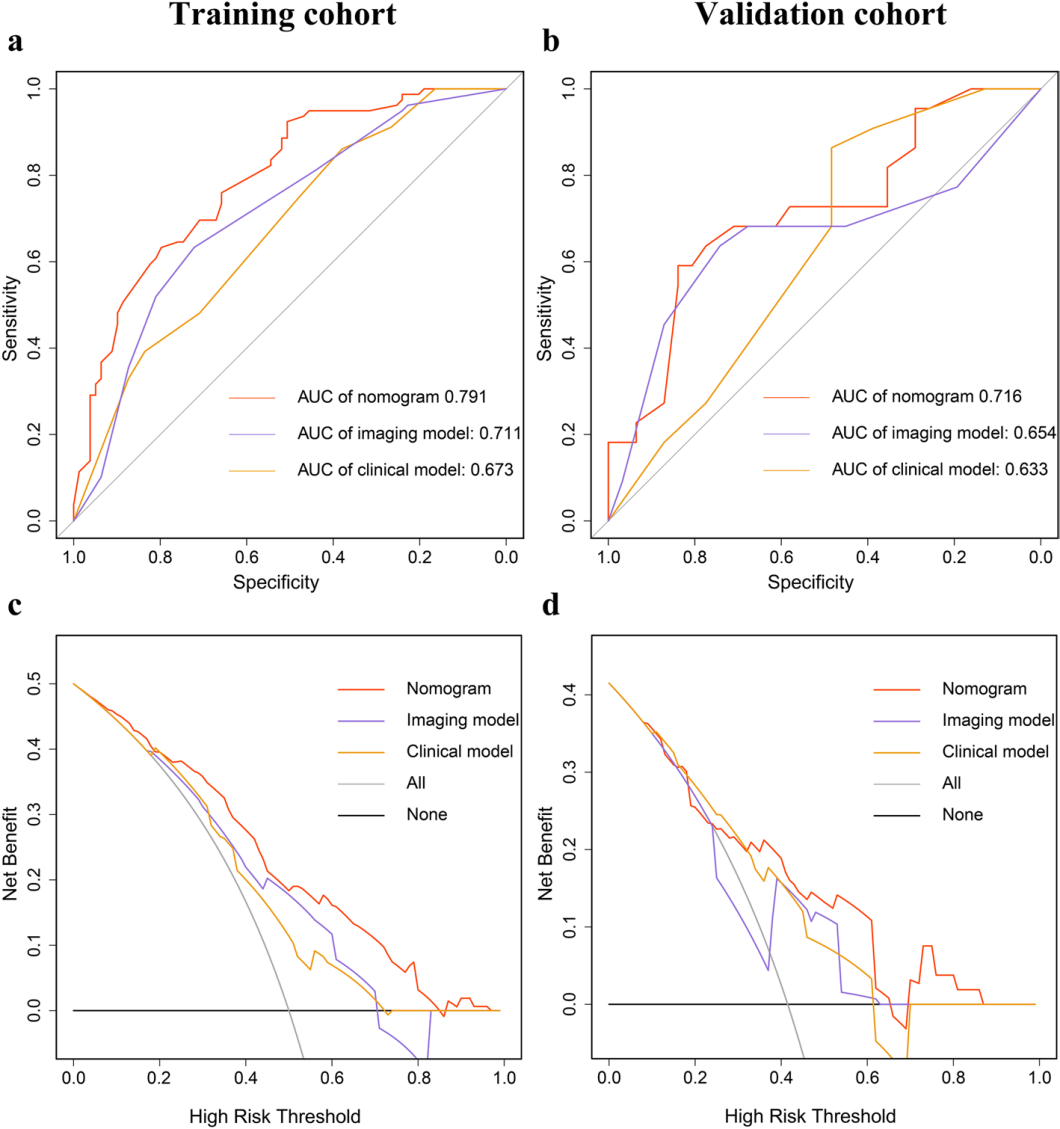
ROC curve (**a**-**b**) and DCA (**c**-**d**) of the nomogram, imaging and clinical models in training and validation cohorts

The independent predictors of MVI included three clinical variables (age, tumor size and cirrhosis) and three imaging variables (VAT density, IMATI and SM area). These clinical and imaging variables were used separately to construct new clinical and imaging model, which were then compared with this nomogram to verify its clinical utility. The ROC curve indicated that the discriminative ability of the nomogram in predicting MVI was better than that of the imaging and clinical models, with AUC values of 0.791, 0.711, 0.673 and 0.716, 0.654, 0.633 in training and validation cohorts, respectively (Fig. 4a, b). In addition, the DCA of nomogram also exhibited a wider range of threshold probabilities in comparison to the imaging and clinical model (Fig. 4c, d).

### Risk stratification of MVI based on the nomogram and survival analysis

**Fig. 5:**
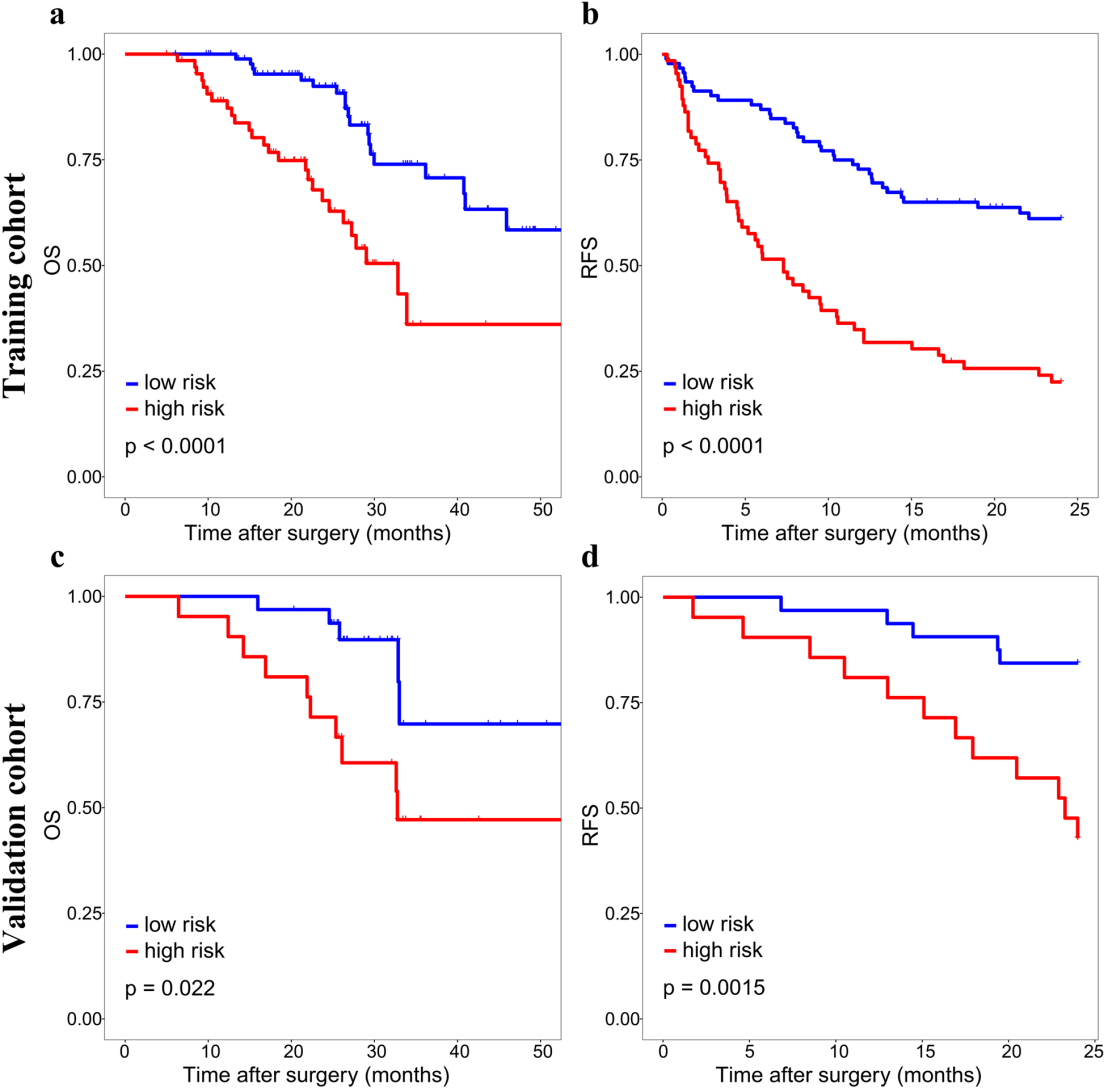
Kaplan–Meier curves of OS and 2-year RFS for patients with different risks scores in training cohort (**a**, **b**) and validation cohort (**c**, **d**)

**Fig. 6:**
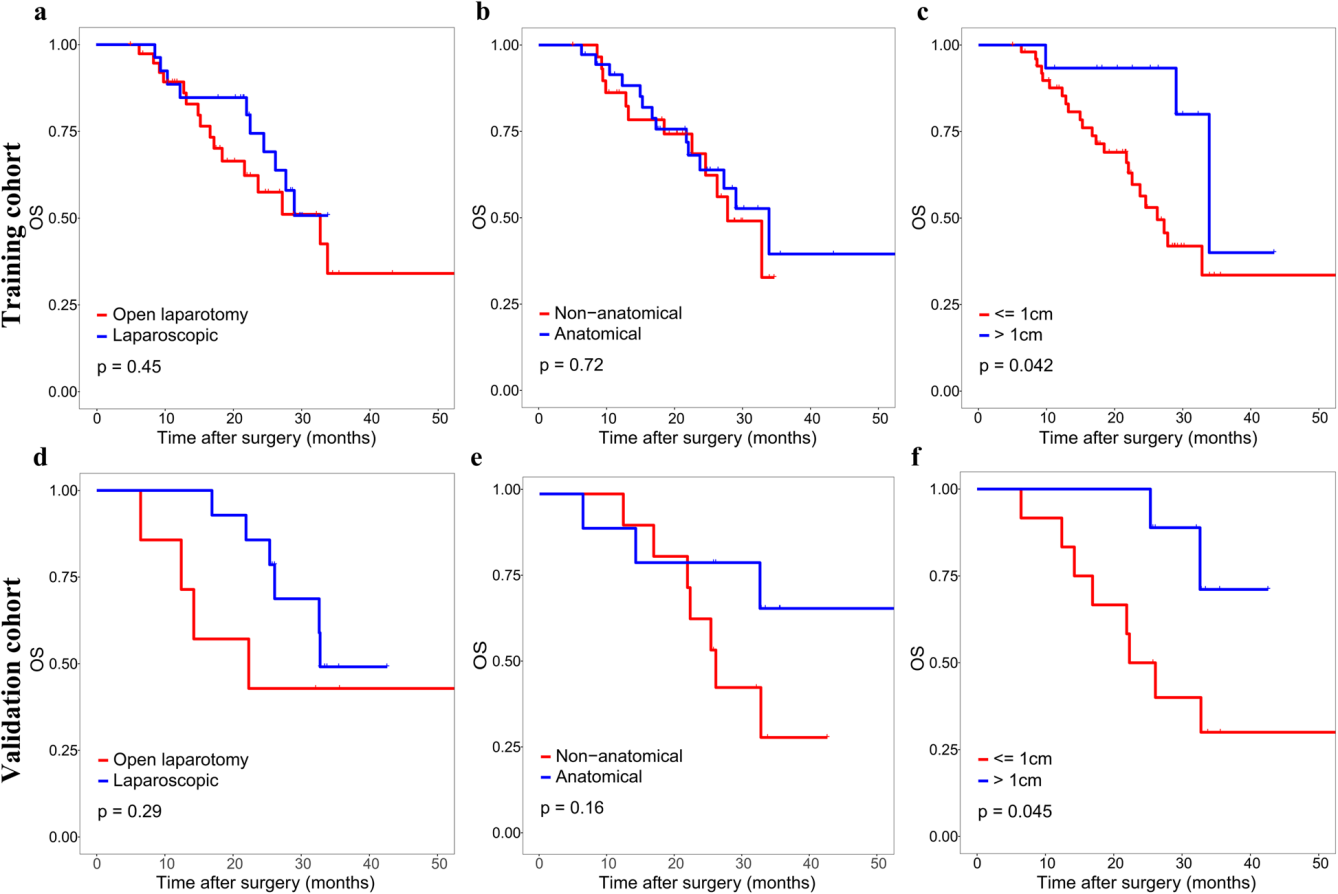
Kaplan–Meier curves of OS for high-risk patients under different surgical approaches, resection methods and surgical margins in training cohort (**a**-**c**) and validation cohort (**d**-**f**)

**Fig. 7:**
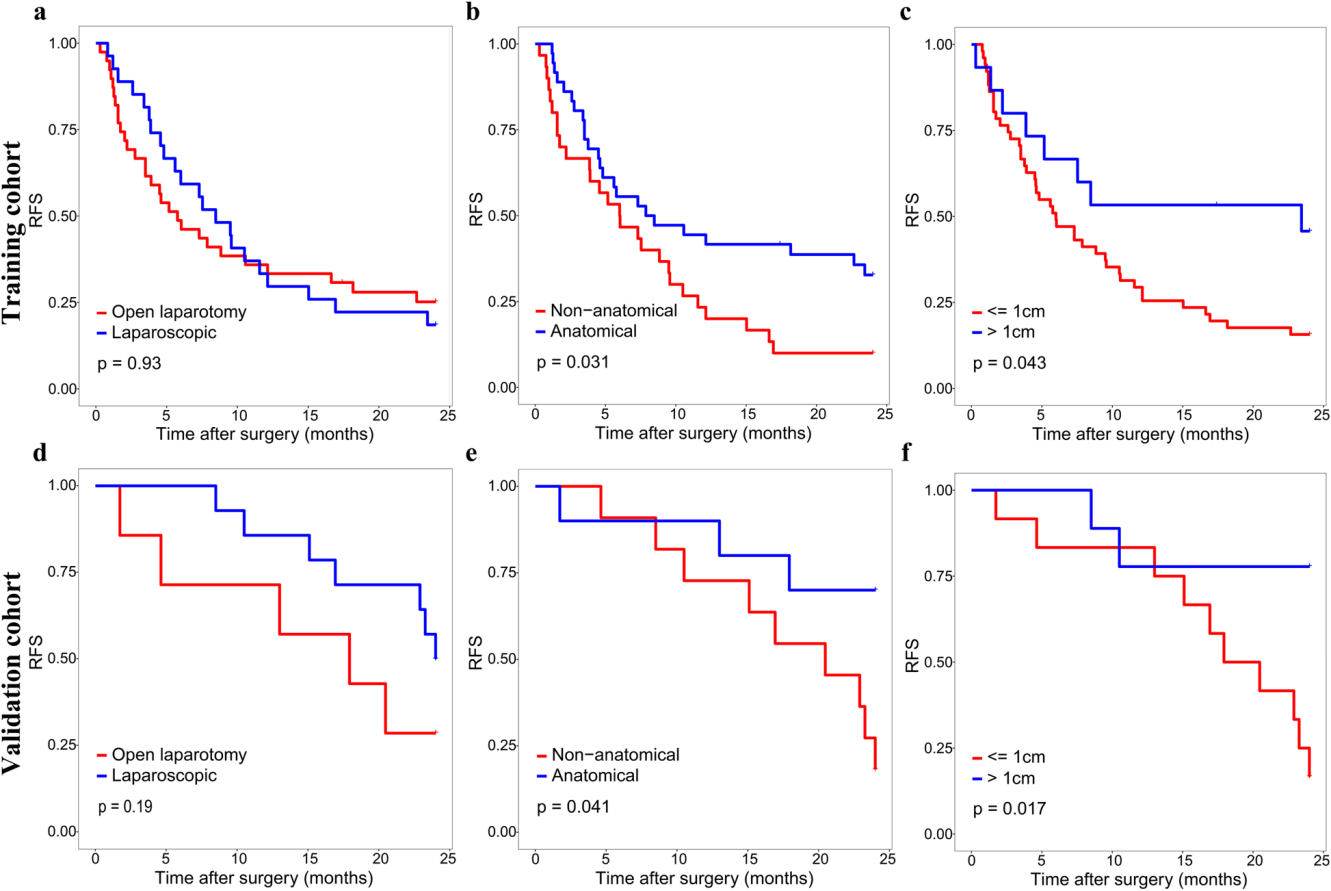
Kaplan–Meier curves of 2-RFS for high-risk patients under different surgical approaches, resection methods and surgical margins in training cohort (**a**-**c**) and validation cohort (**d**-**f**)

The optimal cut-off value of the nomogram scores in training cohort was determined to be 273.8, and the sensitivity and specificity for distinguishing the presence from absence of MVI according to this value were 79.7% and 63.3%, respectively (Supplementary Fig. 2). Based on the cut-off value, patients in two cohorts were divided into high (> 273.8) and low (< = 273.8) risk groups. Kaplan–Meier curves showed that in both training and validation cohorts, patients with high-risk of MVI had higher 2-year recurrence rate and shorter OS than patients with low-risk (Fig. 5). For patients with high MVI risk, wide-margin resection or anatomical resection could significantly improve the 2-year RFS, while only wide-margin resection could significantly improve the OS (Figs. 6 and 7). In contrast, for low-risk patients, neither surgical approaches, nor resection methods or surgical margins had impact on recurrence and OS (Supplementary Figs. 3, 4).

In order to facilitate the clinical use of the nomogram, we have provided an online version accessible via the hyperlink: https://sdmxc.shinyapps.io/MVInom/. By setting variables greater than or equal to the cut-off value to 1 and those less than it to 0, the predicted probability of MVI for patients can be quickly obtained.

## Discussion

In the present study, we showed that adipose and muscle tissues-related variables, including VAT density, IMATI and SM area, were capable to serve as independent predictors for MVI. Furthermore, we incorporated the above variables with three common clinical variables (age, tumor size, cirrhosis) to develop an MVI-predicting model which exhibited good consistency and high accuracy. In the process of validating the clinical utility, our nomogram was also superior to the models only based on imaging or clinical variables. Compared with other radiomics nomograms [11, 12], we only need one plain CT image at L3 level for data extraction, without the need to conduct complex three-dimensional tumor target delineation at various phases of enhanced scanning, which will bring heavy computational burden. Recently, some researchers have employed liver biopsy specimens to analyze gene expression or even radio-genomics for predicting MVI [31, 32]. Despite its good predictive performance, tissue biopsy for the diagnosis of HCC is unnecessary for patients with typical imaging findings according to some clinical guidelines [33, 34]. Additionally, invasive procedure may lead to potential risks such as tumor seeding and fatal bleeding, which outweigh the benefits [34, 35].

In the previously published MVI-predicting nomograms, tumor size, cirrhosis and age has been confirmed as risk factors for vascular invasion in HCC. Pawlik et al. reported that the incidence of MVI was almost twice as high in tumors larger than 5 cm (61%) when compared to smaller tumors (32%), and it increased continuously with increasing tumor size [36]. Cirrhosis patients were frequently present with alterations in hemodynamic and blood microenvironment. On one hand, secondary hypersplenism could decrease platelet counts and reduce portal blood flow to the liver, which was conducive to the formation of MVI [10]. On the other hand, the increase of von Willebrand factor and other procoagulants, as well as the decrease in generation of antithrombin in patients with cirrhosis, could cause an imbalance in the coagulation and fibrinolytic systems [37, 38]. The mechanisms mentioned above could both serve as stimuli for thrombosis and vascular invasion. In current study, younger HCC patients had a higher incidence of MVI, which was in consistent with another research [12]. This observation might be attributed to the high aggressiveness of the tumor in young patients; however, the exact underlying mechanism remains to be investigated by future studies [39].

For body composition-related variables, metabolic theory could support our findings. Because most fatty acids in VAT are transported via the portal vein, higher degree of visceral obesity will expose the liver to high concentrations of fatty acids and glycerol. This, in turn, leads to a series of liver dysfunctions, such as reduced hepatic insulin extraction, increased hepatic glucose production, and ultimately results in type 2 diabetes and insulin resistance [40]. In addition, accumulation of visceral adipose is also linked with the upregulation of aquaporin adipose, an adipocyte-specific glycerol channel, leading to the increased glycerol secretion from VAT [41]. The liver-specific aquaporin protein AQP-9 could absorb excess glycerol and convert it into glycerol phosphate through glycerokinase, which is also one of the mechanisms leading to hyperglycemia [42]. Hyperglycemia alters the size and composition of the basement membranes in micro and large vessels, which in turn increase vascular permeability and fragility, and make it easier for tumors to invasion vascular and metastasize [43–45]. Furthermore, with continues accumulation of lipids, adipose tissues will undergo complex process of remodeling, and the secretion of adipokines, such as leptin, adiponectin, interleukin, vascular endothelial growth factor (VEGF), and hypoxia-inducible factor (HIF) will significantly increase [46], which could induce angiogenesis by stimulating endothelial cell proliferation, migration, and tube formation [47].

SM area provides information on changes in muscle structure, while IMAT indicates alterations in the content of intra- and inter-myocellular adipose [48]. The former parameter denotes muscle quantity and is served as an assessment metric for sarcopenia, and the latter parameter reflects muscle quality and is served as an evaluation index for myosteatosis [20]. Recent studies have established that sarcopenia is positively associated with evaluated all-cause mortality in HCC patients [49, 50]. Though the detailed mechanism has yet to be fully elucidated, however, there are two generally accepted hypotheses. Depletion of muscle could lead to a reduction in the secretion of specific cytokines, such as insulin-like growth factor (IGF)-1, which is associated with advanced clinicopathological variables of HCC [51, 52]. In addition, sarcopenia could also result in the impairment of certain protein hydrolysis systems, such as the tumor necrosis factor (TNF)-α system, thereby facilitating the process of tumor migration and invasion [53].

To date there have no studies that illuminate molecular mechanisms between myosteatosis and tumor angiogenesis. However, pathological changes in muscle have a strong correlation with chronic liver disease [54]. Montano-Loza et al. studied a cohort of cirrhotic patients undergoing liver transplant evaluation, in which the prevalence of muscular steatosis was greater than 50% [55]. In cirrhosis patients, the absorption of lipids and fat-soluble vitamins may be impacted as a result of several physiological changes, such as nausea, anorexia, increased intra-abdominal pressure, and impaired gut motility. Malnutrition promotes the catabolism of muscle proteins, leading to depletion of muscle mass [56, 57]. In addition, chronic hepatic inflammation plays a significant role in promoting systemic inflammation, which in turn also accelerates the catabolism of muscle proteins [58]. There have been studies shown that liver disease not only triggers muscle atrophy and changes its structure, but the muscle in turn promotes the further development of liver disease [54, 59]. Therefore, there may exist an interaction between liver disease and muscle tissue, which exerts an influence on the IMAT, and consequently modulates the expression of downstream adipokines and myokines. Such a dynamic interaction and these cytokines may contribute to the tumor invasion and progression of liver disease.

There are several limitations in our study. Firstly, the limited number of patients impose inherent restrictions on our findings, thus larger external datasets are needed to validate and refine it. Secondly, a prospective study is required to further confirm the reliability of the nomogram. Thirdly, MVI grade was not taken into account in the MVI positive group. Fourthly, this nomogram model is constructed to predict the risk of MVI. Since macrovascular invasion is also a common phenomenon in HCC and is known to be an important predictor of poor survival outcomes [60, 61], whether this nomogram model is fitted for the predication of macrovascular invasion in HCC deserves further study.

In addition, some baseline characteristics showed difference between the two cohorts, and it’s inevitable as the process of data collection in these two hospitals were conducted independently. However, these differences had minimal impact on our results. Firstly, data selection and model construction were both performed within training cohort. Two hundred rounds of internal tenfold cross-validation method was used to verify the stable of our model, which could reduce overfitting of result and maximize the utilization of the data. Secondly, although split sample approach could create two cohorts with no differences [62], it could lead the model being unstable, that is, the performance evaluation was correlated with the partition of the training and validation cohorts. Different partitions might yield different estimates of the accuracy [14, 63]. Therefore, we chose original cohort distribution to ensure the randomness and independence of the data and increase generalizability of the model. In fact, we also verified our model by propensity score matching method to match the baseline characteristics data of the validation cohort and the training cohort, and it still demonstrated good predictive capability (data not shown). This suggests that our model has good generalizability and can be extrapolated to patients in other hospitals.

## Conclusion

By combining 6 preoperative independently predictive factors of MVI, a nomogram is constructed. This model provides an optimal preoperative estimation of MVI risk in HCC patients. For patients with higher MVI scores, anatomical hepatectomy with wide margins may be recommend to reduce early recurrence and extend survival.

### Supplementary Information

**Additional file 1:** **Supplementary Figure 1.** Examples of muscle and adipose tissue measurements: (a) subcutaneous adipose area (b) skeletal muscle area; (c) visceral adipose area; (d) intra-muscular adipose tissue. **Supplementary Figure 2.** The optimal cut-off values of the nomogram scores. **Supplementary Figure 3.** Kaplan-Meier curves of OS for low-risk patients under different surgical approaches, resection methods and surgical margins in training cohort (a-c) and validation cohort (d-f). OS = overall survival. **Supplementary Figure 4.** Kaplan-Meier curves of 2-RFS for low-risk patients under different surgical approaches, resection methods and surgical margins in training cohort (a-c) and validation cohort (d-f). RFS = recurrence free survival. **Supplementary Table 1.** Cut-off values of body composition for male and female.

## Data Availability

All data and material analyzed during this study are included in this article/supplementary file. Further inquiries can be directed to the corresponding author.

## Abbreviations

AFP: Alpha-fetoprotein
ANN: Artificial neural network
BMI: Body mass index
C-index: Concordance index
CT: Computed tomography
DCA: Decision curve analysis
D index: Discrimination index
HCC: Hepatocellular carcinoma
HU: Hounsfield Unit
IMATI: Intramuscular adipose tissue index
IGF: Insulin-like growth factor
KM: Kaplan-Meier
LASSO: Least absolute shrinkage and selection operator
L3: Lumbar vertebra
MRI: Magnetic resonance imaging
MVI: Microvascular invasion
RFS: Recurrence free survival
ROC: Receiver operating characteristic
SM: Skeletal muscle
SMI: Skeletal muscle index
TACE: Transcatheter arterial chemoembolization
TNF: Tumor necrosis factor
VAR: Visceral to subcutaneous adipose tissue area ratio
VAT: Visceral adipose tissue
VEGF: Vascular endothelial growth factor
