# Supplementary material for: A model based on adipose and muscle-related indicators evaluated by CT images for predicting microvascular invasion in HCC patients
Source: Biomark Res. 2023 Oct 4;11:87. doi: 10.1186/s40364-023-00527-z (PMC10548702; doi:10.1186/s40364-023-00527-z)
Supplement: Supplementary file 1 — Additional file 1: Supplementary Figure 1. Examples of muscle and adipose tissue measurements: (a) subcutaneous adipose area (b) skeletal muscle area; (c) visceral adipose area; (d) intra-muscular adipose tissue. Supplementary Figure 2. The optimal cut-off values of the nomogram scores. Supplementary Figure 3. Kaplan-Meier curves of OS for low-risk patients under different surgical approaches, resection methods and surgical margins in training cohort (a-c) and validation cohort (d-f). OS = overall survival. Supplementary Figure 4. Kaplan-Meier curves of 2-RFS for low-risk patients under different surgical approaches, resection methods and surgical margins in training cohort (a-c) and validation cohort (d-f). RFS = recurrence free survival. Supplementary Table 1. Cut-off values of body composition for male and female. [file 40364_2023_527_MOESM1_ESM.doc]

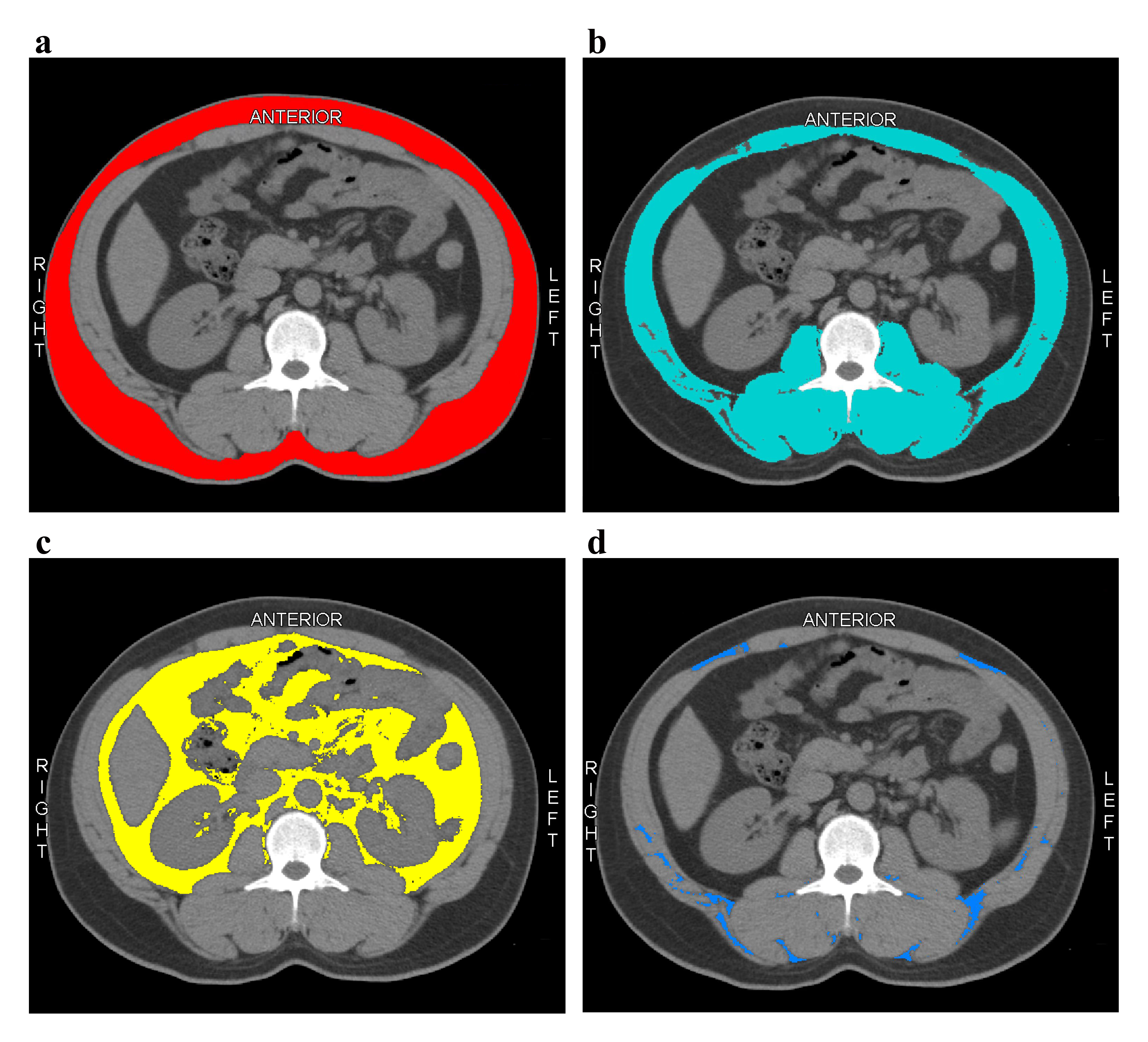


Supplementary Figure 1. Examples of muscle and adipose tissue measurements: (a) subcutaneous adipose area (b) skeletal muscle area; (c) visceral adipose area; (d) intra-muscular adipose tissue


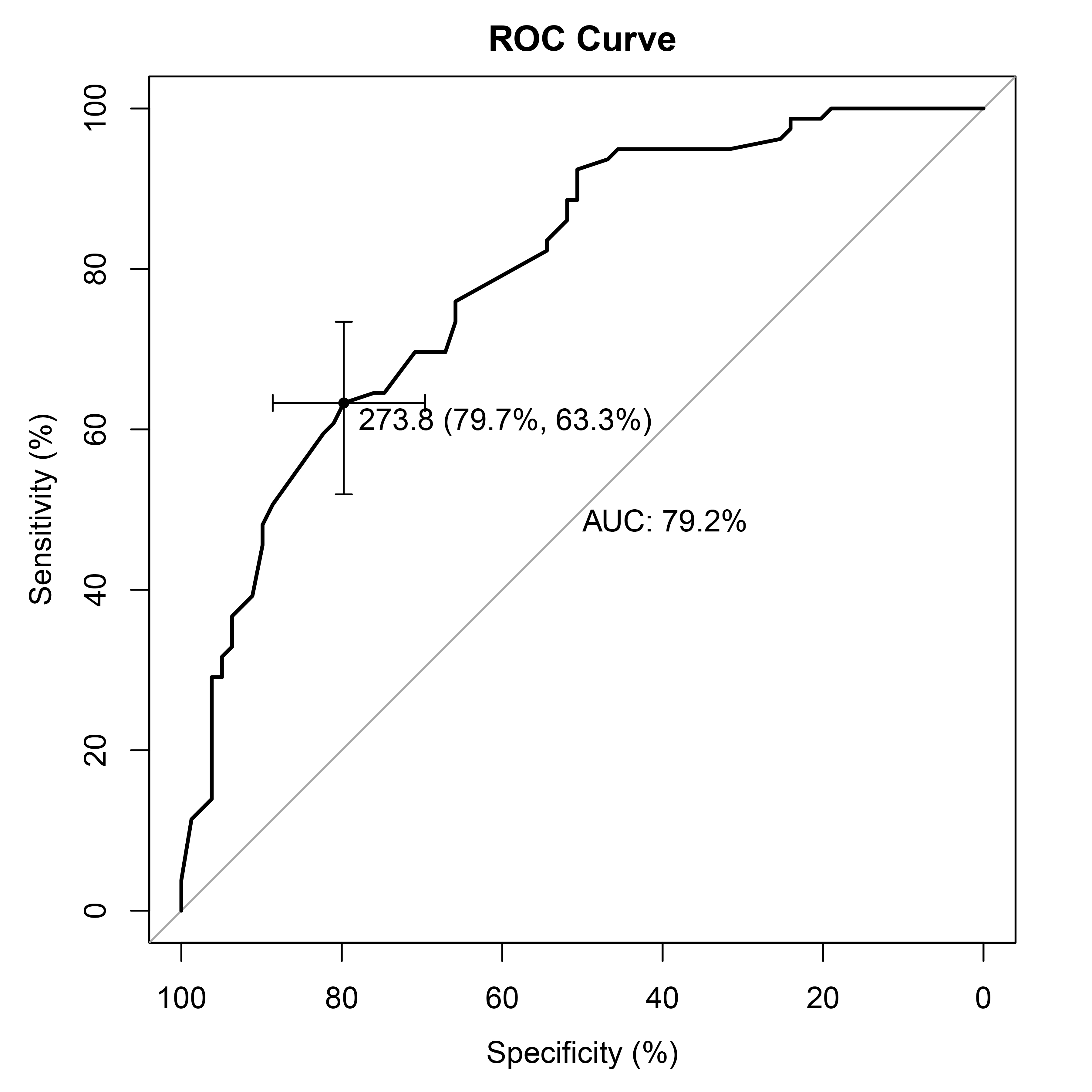


Supplementary Figure 2. The optimal cut-off values of the nomogram scores.


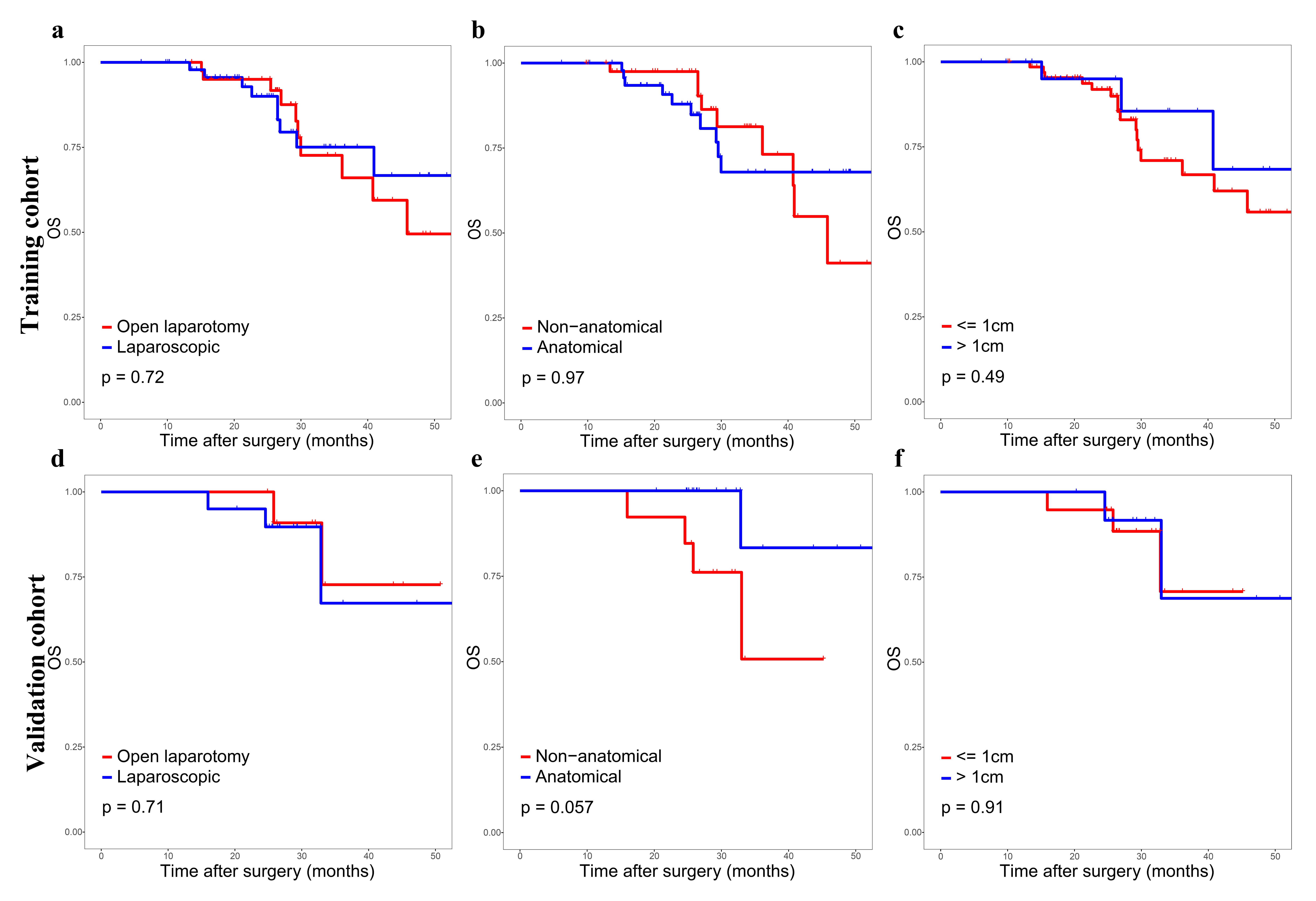


Supplementary Figure 3. Kaplan-Meier curves of OS for low-risk patients under different surgical approaches, resection methods and surgical margins in training cohort (a-c) and validation cohort (d-f). OS = overall survival.


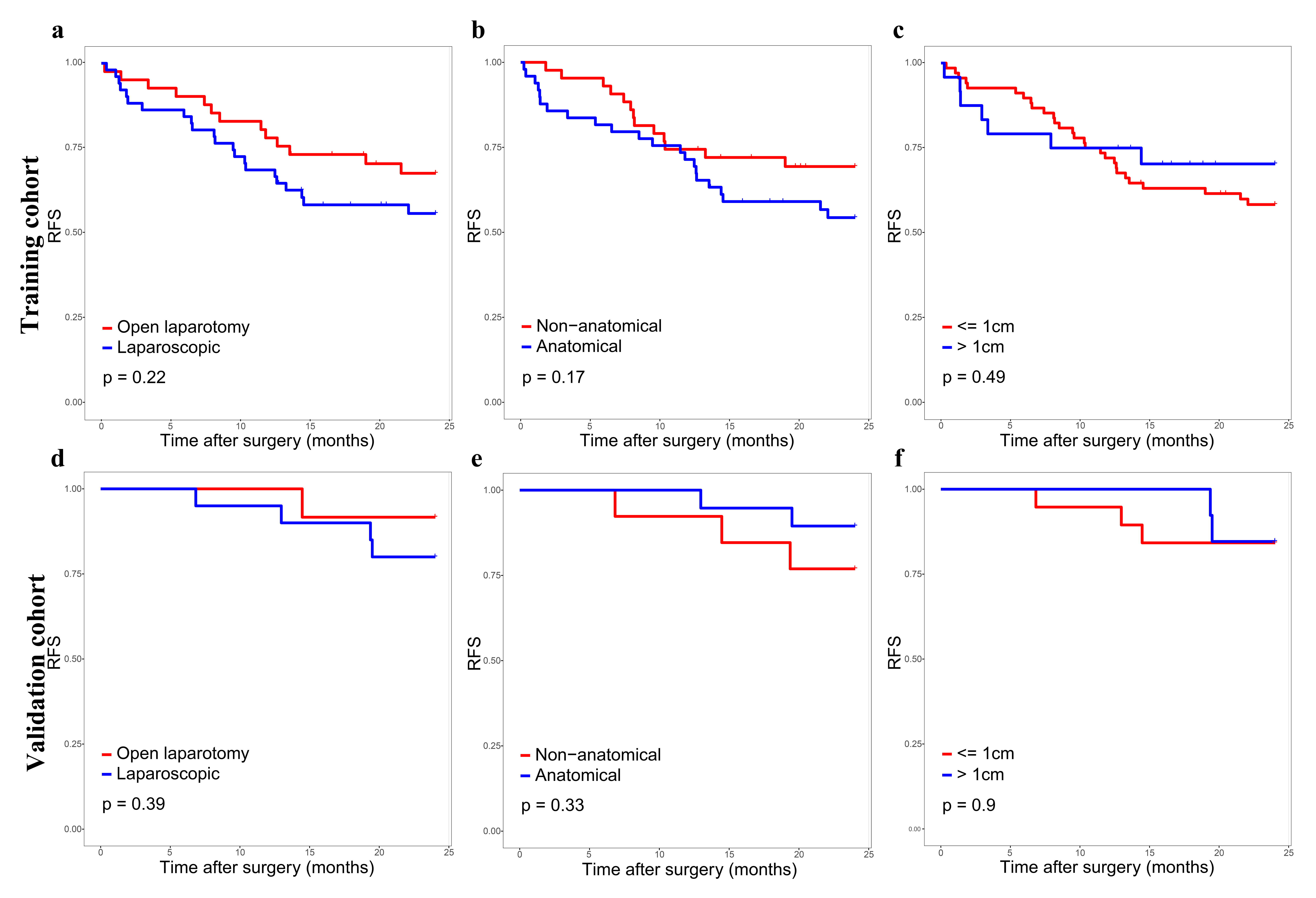


Supplementary Figure 4. Kaplan-Meier curves of 2-RFS for low-risk patients under different surgical approaches, resection methods and surgical margins in training cohort (a-c) and validation cohort (d-f). RFS = recurrence free survival.

Supplementary Table 1. Cut-off values of body composition for male and female

| Variables | Male | Female |
| --- | --- | --- |
| SAT area (cm^2^) | 102.73 | 87.03 |
| SAT density (HU) | -99.16 | -104.45 |
| SATI | 47.95 | 104.41 |
| VAT area (cm^2^) | 121.58 | 91.73 |
| VAT density (HU) | -91.46 | -85.62 |
| VATI | 39.78 | 39.98 |
| VSR | 1.00 | 0.67 |
| IMAT area (cm^2^) | 10.99 | 10.82 |
| IMAT density (HU) | -66.57 | -68.62 |
| IMATI | 3.70 | 4.23 |
| SM area (cm^2^) | 131.05 | 96.48 |
| SM density (HU) | -40.35 | -35.60 |
| SMI | 49.44 | 37.67 |

Abbreviations: HU, Hounsfield Unit; SAT, subcutaneous adipose tissue; SATI, subcutaneous adipose tissue index; VAT, visceral adipose tissue; VATI, visceral adipose tissue index; VSR, visceral to subcutaneous adipose tissue area ratio; IMAT, intramuscular adipose tissue; IMATI, intramuscular adipose tissue index; SM, skeletal muscle; SMI, skeletal muscle index.
